# Atomic Force Microscopy (AFM) on Biopolymers and Hydrogels for Biotechnological Applications—Possibilities and Limits

**DOI:** 10.3390/polym14061267

**Published:** 2022-03-21

**Authors:** Jnanada Joshi, Sarah Vanessa Homburg, Andrea Ehrmann

**Affiliations:** Faculty of Engineering and Mathematics, Bielefeld University of Applied Sciences, 33619 Bielefeld, Germany; jnanada_shrikant.joshi@fh-bielefeld.de (J.J.); sarah_vanessa.homburg@fh-bielefeld.de (S.V.H.)

**Keywords:** nanoindentation, elastic modulus, peak force quantitative nanomechanical mapping, KPFM, interaction forces, adhesion, impedance, adsorption, ultracentrifugation, drop deposition

## Abstract

Atomic force microscopy (AFM) is one of the microscopic techniques with the highest lateral resolution. It can usually be applied in air or even in liquids, enabling the investigation of a broader range of samples than scanning electron microscopy (SEM), which is mostly performed in vacuum. Since it works by following the sample surface based on the force between the scanning tip and the sample, interactions have to be taken into account, making the AFM of irregular samples complicated, but on the other hand it allows measurements of more physical parameters than pure topography. This is especially important for biopolymers and hydrogels used in tissue engineering and other biotechnological applications, where elastic properties, surface charges and other parameters influence mammalian cell adhesion and growth as well as many other effects. This review gives an overview of AFM modes relevant for the investigations of biopolymers and hydrogels and shows several examples of recent applications, focusing on the polysaccharides chitosan, alginate, carrageenan and different hydrogels, but depicting also a broader spectrum of materials on which different AFM measurements are reported in the literature.

## 1. Introduction

The topography, roughness and similar morphological parameters of surfaces are often investigated by microscopic methods. These parameters are not only important in materials sciences, during the development of new materials with different surface morphologies, but also in biotechnology and many other research areas where a substrate’s surface plays an important role for the adhesion of other materials or living cells, etc.

While light microscopy has been well-known for hundreds of years [1], electron microscopy has not even been used for a century now [2], and AFM was first mentioned only in 1986 [3]. Since then, it has rapidly had more and more impact in microscopy techniques, as depicted by Parot et al. in their review of the history of AFM in the life sciences [4]. In 1987, the first measurements in liquid were performed, the first membrane proteins were depicted in 1991, in 1997 the first single protein unfolding was observed, and in 2004, the native organization of membrane protein supercomplexes were reported, etc. From year to year, technical innovations have been added, such as new cantilevers, the tapping mode often used on biological samples, high-speed techniques, and many more [4], leading to the recent state in which many biological and biotechnological research groups use an AFM as naturally as a fluorescence microscope. The main advantage of an AFM against light microscopes is its resolution which may reach that of single atoms on a flat sample surface for inorganic matter, while the AFM of functional biomolecules in aqueous solutions has been shown to achieve a resolution around 1 nm, which is identical to the smallest tip radius [5]. For a less powerful resolution, AFM images can be taken in the air or in liquids, making it also advantageous for biological—usually water-containing—tissue in comparison to scanning electron microscopy (SEM) which mostly needs a vacuum in the sample chamber [6].

Besides the pure topographic information, however, scanning a sample with a cantilever tip by measuring the forces between the tip and the sample allows for detecting even more information—such as the elastic modulus, hardness, friction, surface charges, adhesion between the tip and sample, and others. These additional measures are enabled by different measurement modes, functionalized tips and sophisticated evaluation methods.

This review is structured as follows. In Section 2, we present an overview of different basic AFM modes, such as contact, tapping and noncontact, and modes giving more information than the pure surface nanostructure, e.g., by nanoindentation, conductive AFM, peak force quantitative nanomechanical mapping, Kelvin probe force microscopy (KPFM) and others, which are often applied to investigate biopolymers as films or hydrogels in air and in liquids. In Section 3, we describe in detail the biopolymers chitosan, alginate, and carrageenan as well as the synthetic polymer silica, that are most commonly applied in biotechnology and biomedicine which are in the focus of this review. Section 4 starts with a short overview of possible sample preparation methods, followed by typical AFM measurements of the chosen biopolymers as well as the silica and biopolymer hydrogels. At the end of Section 4, a brief tabular overview of the AFM measurements on many other biopolymers and hydrogels relevant for interaction with biological material is given.

## 2. AFM Techniques

**Figure 1:**
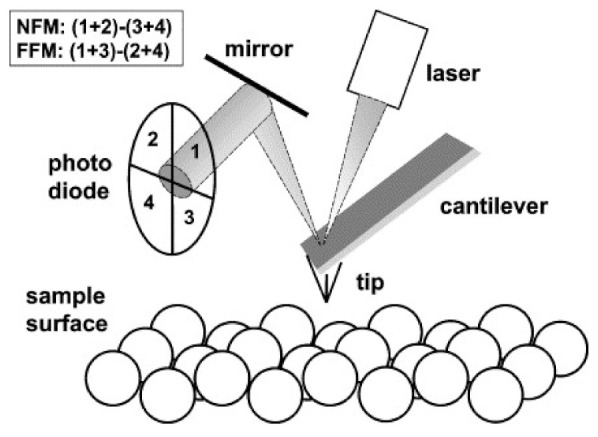
AFM operation principle. The cantilever with its tip is deflected by the sample surface topography, which is detected with a laser-optical set-up. The photodiode measures normal forces (normal force microscopy, NFM) and frictional forces (FFM) moving the tip. The piezotube scanner allowing scanning motions in *x*- and *y*-directions as well as moving in *z*-direction is not shown here. Reprinted from [7], Copyright (2001), with permission from Elsevier.

The atomic force microscope is recently the most often used scanning probe microscope. Its general working principle can be described as follows (Figure 1) [7]: a tip, often produced from Si or Si_3_N_4_ and with a typical tip radius around 1 nm to 20 nm, sometimes larger, is attached to the end of a cantilever. The cantilever can vibrate with a specific spring constant, typically with frequencies around some ten to a few hundred kilohertz. By measuring the signal on a photodiode, the z-position of the cantilever holder is usually moved so that a constant force between the tip and the surface is maintained. This moving z-position is transferred into the surface topography.

The forces between the tip and the surface are mostly—without an additional functionalization of the tip—based on electrostatic Coulomb forces (repulsive) and van der Waals forces (attractive) and are exceedingly small, approx. in the range of 10^−11^ N to 10^−7^ N. The distinctly small distances between the tip and surfaces—typically around 0.1 nm to 10 nm—enable a resolution in the order of 0.1 nm under perfect conditions, especially in the case of a perfectly smooth surface. Other forces, however, may superpose the aforementioned ones and have to be taken into account during the interpretation of AFM images [7].

### 2.1. Topography and Roughness

**Figure 2:**
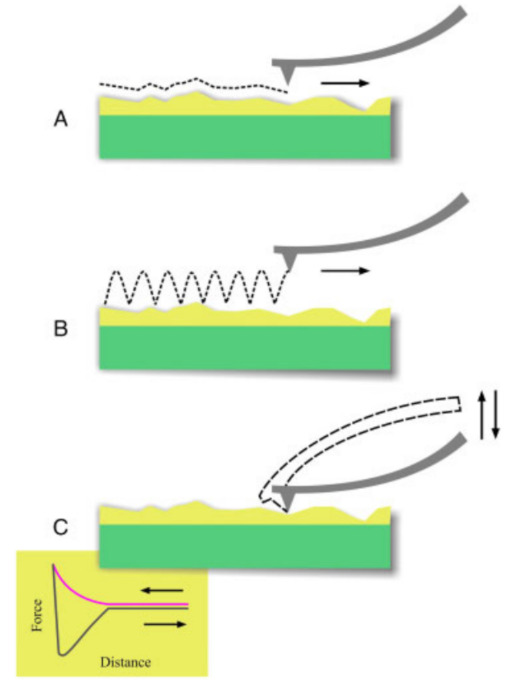
AFM operating modes: (**A**) contact mode; (**B**) dynamic mode for topographic imaging (the oscillation frequency in the dynamic mode is much higher than that in the scheme, i.e., the tip oscillates many times per pixel); and (**C**) force spectroscopy mode for interaction probing. Reprinted from [8], Copyright (2010), with permission from Wiley.

The cantilever can approach the sample surface in different ways—either in the contact mode (Figure 2A) or in the dynamic (tapping) mode (Figure 2B) [8]. While the resolution can be higher in the contact mode, the lateral forces due to the friction between the tip and sample may be problematic for soft or uneven surfaces, which is why for such samples often the dynamic mode is chosen. This means that the cantilever performs oscillations near its resonance frequency and only taps towards the sample briefly, so that the lateral movement can be performed without the tip sticking to the sample surface [8]. It should be mentioned that in the so-called noncontact mode, the cantilever also vibrates, but with a smaller amplitude, thus not touching the sample surface. Yang et al. reported that the noncontact mode exerts a delicate force on the sample at the cost of less precise height measurements, while the tapping mode caused deformations of the soft materials, especially in liquid environments [9]. Such topography investigations have been performed on many different materials during the last decades, e.g., on food biopolymers [10], on biopolymer networks [11], proteins and DNA [12,13], or hydrogels [14].

Topography measurements also allow calculating the surface roughness, which is especially interesting for homogeneous, isotropic surfaces, while anisotropic morphologies—e.g., from nanofibers or fibroblasts—usually give more information in the full topography image.

By measuring the cantilever deflection, which is correlated to the force on the tip, during vertical displacement, it is also possible to measure force-distant curves (Figure 2C) in the so-called force spectroscopy mode, which allows for detecting interaction forces between the tip and sample as well as an elastic modulus (Young’s modulus) which may vary from the values from macroscopic tests, but is actually supportive for the evaluation of microorganisms, nanoparticles, hydrogels or other small-scale samples [8]. The force-distance curve shows an increasing force during approaching (red part of the curve), based on electrostatic and van der Waals forces. During retraction, firstly the tip sticks to the surface due to adhesive forces, leading to a strong decrease in force, i.e., a negative cantilever deflection, before it becomes free and the force approaches the original one again.

**Figure 3:**
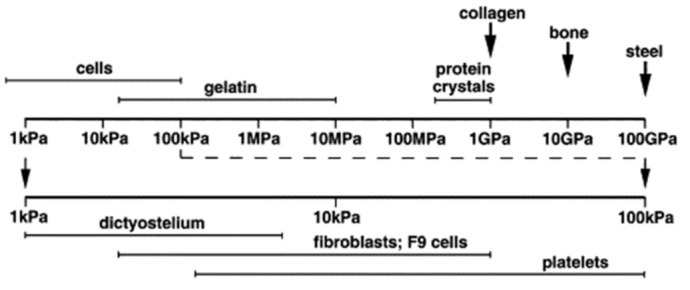
Elastic moduli of different biological and other materials. Reprinted from [15], Copyright (2003), with permission from Elsevier.

Generally, such forces are quite small for cells, tissue, soft hydrogels and biopolymers in different forms, as depicted in Figure 3 [15]. It should be mentioned that by functionalizing the AFM tip with specific groups or molecules, even more information can be gained from the interaction between the sample and the functionalized tip [16].

### 2.2. Phase Imaging

Phase imaging belongs to the standard modes of an AFM and is thus usually also recorded when topography images are taken in the tapping mode. The corresponding images show the phase between the piezo-driven excitation and the actual oscillation of the cantilever. Since this phase depends on the sample hardness, elasticity and adhesion, it allows for distinguishing different materials in a sample; however, only in a qualitative way [17].

**Figure 4:**
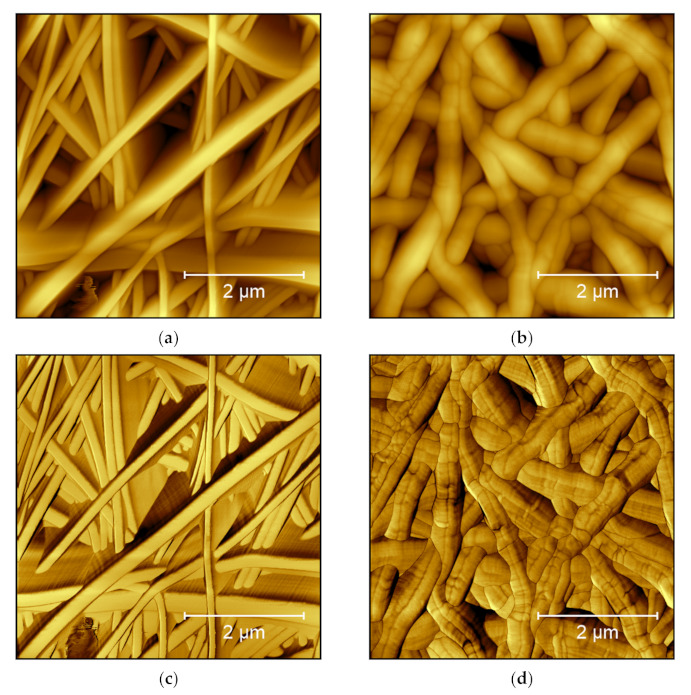
AFM images taken on poly(acrylonitrile) (PAN) nanofiber mats: (**a**) raw mat, topography image; (**b**) hot-pressed mat, topography image; (**c**) raw mat, phase image; and (**d**) hot-pressed mat, phase image. Images taken with a Nanosurf FlexAFM with the following scanning parameters: 512 points × 512 lines, 1.4 s/line, setpoint 55%, p-gain 550, i-gain 1000, d-gain 100, free vibration amplitude 6 V.

Moreover, the phase image increases the visibility of edges, sometimes making small features more visible [7]. This is especially interesting for highly irregular surfaces, where the feature height necessitates relatively large free vibration amplitudes of the cantilever which lead to a lower noise level, but also lower the resolution. As an example, from our own research, Figure 4 shows AFM measurements under identical conditions on two poly(acrylonitrile) (PAN) nanofiber mats after electrospinning (Figure 4a) and after hot-pressing at 180 °C (Figure 4b). These topography maps show that after hot-pressing, the fibers become thicker, as expected. In Figure 4a, however, the fibers look more like high walls, which is not possible. This artifact is not visible in the phase image of the raw nanofiber mat (Figure 4c), where the areas between the clearly separated fiber surfaces show different phases. This finding can be attributed to small movements of the nanofibers during scanning, making them look broader in the topography image. On the other hand, Figure 4d clearly shows dark lines in the phase map of the hot-pressed sample. These lines are not related to the aforementioned material variations, as they are also slightly visible in the topography (Figure 4b), but they show topographical constrictions upon heating, as they are also known from the thermal stabilization of PAN nanofiber mats [18,19].

Besides this relatively simple technique, many more modes can be applied with most modern AFM instruments, partly with modified equipment and often applying specialized cantilevers, partly by simply changing the measurement parameters. On the other hand, small changes in the measurement situation can have a large impact on the results, as the next section shows.

### 2.3. Attractive and Repulsive Interaction Regimes

**Figure 5:**
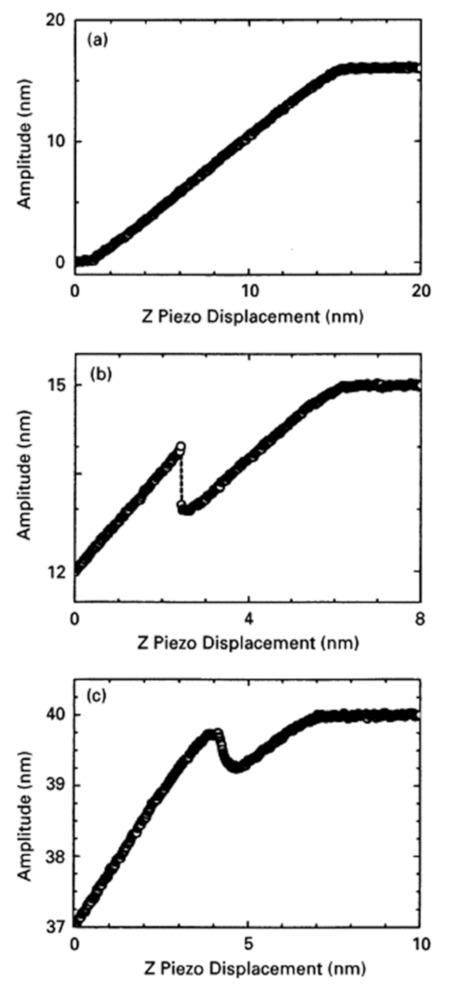
Measured amplitudes vs. z-displacements between tip and sample surface for (**a**) mica (free amplitude 16 nm); (**b**) InAs/GaAs (free amplitude 15 nm); and (**c**) InAs/GaAs (free amplitude 40 nm). Reprinted from [22], Copyright (2000), with permission from Elsevier.

In the tapping mode, the cantilever oscillated near its resonance frequency, and the amplitude reduction due to the forces between tip and surface is measured. This amplitude modulation feedback is influenced by attractive as well as by repulsive forces. This means, on the one hand, that a distance where parts of the sample show repulsive forces of similar dimension as the attractive forces in other sample parts will lead to a low contrast as no differentiation between both is possible [20]. García and San Paulo calculated the discontinuities in the amplitude and phase shift curves for crossing the border between both regimes [21] and described different possibilities for how the amplitude could be related to the displacement between the tip and sample, measured on different samples with varying free amplitudes (Figure 5) [22]. In case (a), e.g., the amplitude is insensitive to displacements for larger distances than approx. 15 nm, while a linear correlation is found for smaller distances. In both other cases, however, there is a local maximum which they attribute to the competition of different interaction regimes, i.e., long-range attractive and short-range repulsive forces, causing possible problems in the interpretation of the corresponding topography images.

The differentiation between two interaction regimes, however, can not only cause problems, but also be used to choose the optimum regime for a specific measurement. Round and Miles, e.g., report a contrast change in topography and phase images during measurements on DNA in air, which they attributed to a nonlinear dynamic response of the cantilever near the surface, i.e., near a repulsive barrier [23]. They showed that by slightly modifying the driving frequency around the resonance frequency, the topography and phase contrast could be varied and even switched off. San Paulo and García mentioned that in the repulsive regime, usually the contrast and resolution were reduced, combined with damaging the tip due to the forces between the tip and the sample [24].

An interesting point was raised by Zitzler et al. who investigated the influence of relative humidity on measurements on hydrophilic samples [25]. They showed that generally an adsorbed water layer on the sample surface could interact with the tip due to capillary forces. When the cantilever oscillates with sufficiently high amplitude near the sample surface, a capillary neck could be formed between the tip and the sample, leading to a hysteresis in the force-distance curve which may especially be important for samples with locally varying wettability, as often found in biological samples. This effect was also shown for contact-mode measurements [26,27].

Maragliano et al. used the transition between attractive and repulsive force regimes for the evaluation of the tip radius [28]. They showed that for a sharper tip, the value of the free amplitude leading to a transition between these regimes was smaller. On the other hand, they used capacitance-distance curves combined with an analytical model. The first method, measuring the minimum critical amplitude to reach the border between both regimes, i.e., bistable behavior as shown in Figure 5b,c, was found to give more accurate results especially for fine tips.

### 2.4. Nanoindentation

**Figure 6:**
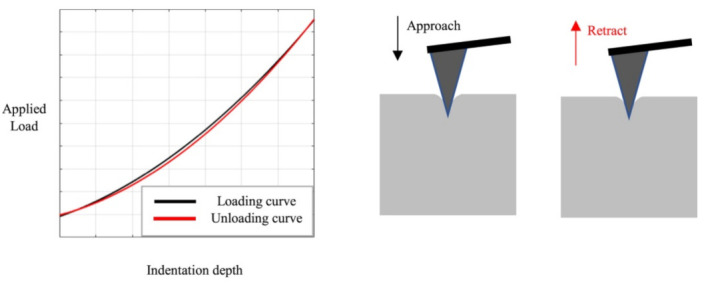
Indentation experiment with loading and unloading curves. Reprinted from [29], originally published under a CC-BY license.

Nanoindentation in the AFM can be used to evaluate the mechanical characteristics of biological and other samples. Such experiments are performed by indenting the AFM tip into the sample and retracting it again, leading to load-indentation curves, as shown in Figure 6 [29]. The authors describe different possibilities for the evaluation of such experiments, i.e., the most often used Hertz model and the Oliver and Pharr analysis, and evaluate the influence of the indenter shape. They found clear differences for not perfectly elastic samples and suggested the Hertz model only in this case. Recently, Kontomaris et al. discussed an extension of the Hertz model for biological samples including indentation depth, tip radius, and sample shape to overcome problems with the common Hertz model [30].

Qian and Zhao reviewed nanoindentation especially for soft biological materials [31]. They mentioned that for soft biological samples, relatively soft tips could be used, such as silica or silicon, aluminum or steel in addition to the typical materials for nanoindentation such as sapphire or diamond. Similarly, they found large tips in the range of millimeters being used for particularly soft biomaterials and suggested using a tip size much smaller than the tissue and much larger than an individual cell or fiber in the case of tissue-level experiments. Similar materials and dimensions as well as different tip shapes were mentioned by Vlassov et al., reviewing nanoindentation experiments on polydimethylsiloxane (PDMS), a silicon-based organic polymer often used in microfluidics and other areas [32].

Sokolov et al. mention for the special case of nanoindentation on eukaryotic and Gram-negative prokaryotic cells that the brush surrounding them has to be taken into account, and that these experiments enabled measuring the length and grafting density of the brush [33]. They formulated some rules regarding nanoindentation on cells, such as working only on the flat part of a cell, keeping the vertical ramping speed constant, checking the linearity of the mechanical response of the cell body material, and collecting enough data per cell for a proper statistical treatment.

Finally, Guo and Roos showed that nanoindentation on protein shells was even possible in liquids, i.e., similar to the physiological environment [34].

### 2.5. Peak Force Quantitative Nanomechanical Mapping (PeakForce QNM)

Another method to determine the elastic modulus of a sample is the PeakForce QNM technique. The PeakForce QNM tapping mode works similar to the normal tapping mode, with the difference that in the common tapping mode the cantilever vibration amplitude is kept constant, while in the peak force mode the maximum force is controlled to avoid damage of the tip or sample [35]. They described measuring the architecture of an intact plant cell in fluid by this technique.

Dokukin and Sokolov compared PeakForce QNM measurements on polyurethanes and polystyrene with results from nanoindentation and other techniques and found that, for sharp probes, the PeakForce QNM measurements overestimated the elastic modulus, while dull AFM probes with tip diameters around 240 nm could be used for quantitative mapping of the elastic modulus with a resolution of approx. 50 nm [36].

**Figure 7:**
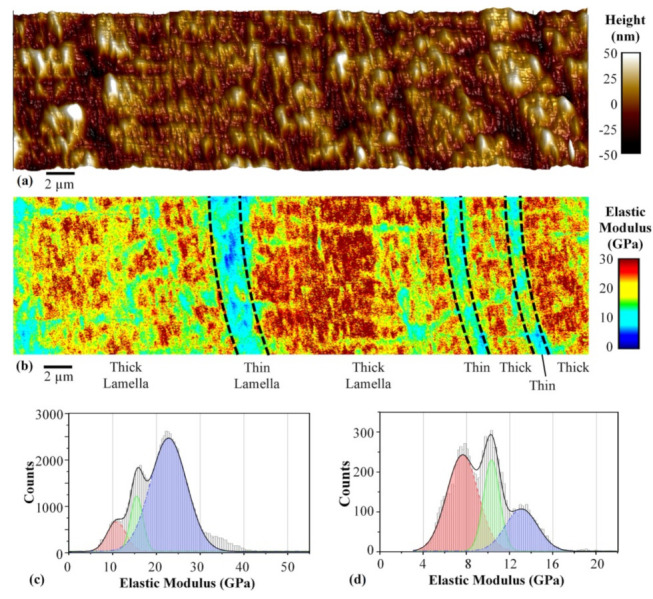
AFM measurement of a bovine cortical bone: (**a**) topography, (**b**) elastic modulus map, with stitched scans showing thick and thin sub-lamellae in an osteon; and measured and modeled histograms of elastic moduli for (**c**) thick sub-lamellae and (**d**) thin sub-lamellae. Reprinted from [37]. Copyright (2020), with permission from Elsevier.

Zhou et al. used the PeakForce QNM to investigate bovine cortical bones in water [37]. They found differences in the elastic moduli of osteons, interstitial bones, cement lines and different sub-lamellae and could visualize soft mineralized collagen fibrils in a harder matrix, as depicted in Figure 7 [37]. The elastic moduli were measured and modeled (Figure 7c,d), based on the Derjaguin–Muller–Toporov (DMT) model with a Hertzian contact profile which was used to fit the load-deformation curves for the tip retraction process. DMT modeling was also used by Schön et al. for mapping the elastic moduli in phase-separated polyurethanes [38]. Generally, for systems with low adhesion and small tip radii, the DMT theory is advantageous, while the Johnson–Kendall–Roberts (JKR) theory is used for highly adhesive systems with low stiffness and large tip radii, and the Hertz theory is only suitable for negligible adhesion forces [36].

### 2.6. Hardness Measurements

Hardness measurements on different samples can be performed, similar to elasticity measurements, by nanoindentation. Here again, the indenter shape and the tip radius have to be taken into account during the interpretation of the measured curves. Calabri et al. calculated correction factors for different tip curvature radii [39]. They mentioned that the tip radius is less important for the indentation process itself, but for the subsequent imaging process of the area impressed during indentation. These findings could also be used to model the effect of worn tips on nanoindentation correctly. With these corrections, hardness measurements by AFM only slightly underestimated the hardness in comparison with typical literature values.

While some groups investigated the special challenges related to thin-film systems and rough surfaces, e.g., by reducing the necessary indentation depth [40], for biomaterials it is often more important that working under water is possible. This was shown, e.g., by Balooch et al. who compared hardness and elastic modulus measurements of demineralized human dentin in water, in air after desiccation and in water after rehydration [41]. They found strong differences between the viscoelastic values and elastic moduli measured in water and after desiccation, while rehydration did not fully bring the original values back, which was attributed to oxidation-induced crosslinking and chain entanglement of the collagen upon drying. It must be mentioned that the fully hydrated dentin specimens did not have a measurable hardness value since it was impossible to reach a permanent deformation by nanoindentation.

### 2.7. Adhesion Measurements

For adhesion measurements, many authors report on using cantilevers with an increased adhesion area, e.g., by using an elastomeric colloidal probe [42]. Erath et al. applied the aforementioned Johnson–Kendall–Roberts (JKR) approach, measuring the contact area as a function of load combined with elastic parameters, to distinguish between the capillary forces in air, hydration forces and hydrophobic interactions in water for the interaction between the soft colloid and the substrate.

Dong et al. investigated the adhesion between a protein and a TiO_2_ substrate by using a lysozyme-modified tip [43]. The adhesion was measured by the force jump upon retraction, i.e., identified as the pull-off force necessary for separation of the tip from the surface. Similarly, Wojcikiewicz et al. attached 3A9 cells by concanavalin A-mediated linkages to an AFM cantilever, approached the sample until the cell was in contact with the surface, and retracted the cantilever with the cell until separation, in this way measuring the detachment force [44]. The adhesion between the DNA and living cells was measured in a similar way by Hsiao et al. using DNA-coated cantilevers and measuring the de-adhesion force during retraction of the cantilever [45].

Besides the topological and mechanical properties described in the previous sections, it is also possible to measure the chemical or electronic properties of specimens. Some of the techniques related to biopolymers and hydrogels are described in the next sections.

### 2.8. Kelvin Probe Force Microscopy

Kelvin probe force microscopy (KPFM) allows for mapping the surface potential of a sample [46]. This electrical AFM mode measures the difference in work function or contact potential difference between the tip and the sample surface [47]. The work function defines the minimum energy necessary for an electron to leave the surface of a material. While the work function is often mentioned in correlation with metals, biomolecules also have a work function, i.e., an energy difference between the “outer” electron in the sample and the vacuum level [48]. It is influenced by local electromagnetic and also mechanical properties, such as surface charges, dielectric constants or the doping level of a semiconductor.

Generally, KPFM is measured with conductive probes whose work function can be calibrated on highly-oriented pyrolytic graphite (HOPG) for which the work function in air is well-known. When the AFM tip is near the sample surface, an electrical force occurs due to the difference in Fermi levels [49]. Leveling out this additional electrical force by an external bias voltage (combining an AC and a DC signal) enables calculating the contact potential difference and thus the material’s work function. It is still necessary to distinguish between the electrical and topography signal, which can be completed with a high-frequency AC voltage combined with a sophisticated evaluation, depending on the measurement mode (frequency modulation or amplitude modulation measurement).

While some groups work on measuring and interpreting highly resolved KPFM images on the atomic scale [50], for biomaterials the KPFM measurements in liquid are more interesting, which are indeed possible, as described by Collins et al. [51].

### 2.9. Conductive AFM

Another electric measurement type is conductive AFM or conducting probe AFM, also called C-AFM or CP-AFM. It can be used, e.g., to measure the varying thickness of an oxide film on a semiconducting substrate [52] or the resistance of semiconducting nanowires [53].

Molecular crystals from sexithiophene were investigated by CP-AFM, using Au-coated Si probes to reduce the contact resistance [54]. They found resistances of a few hundred MΩ along some hundred nanometers, with significantly higher resistances around a few hundred GΩ if the measurement was performed via a grain boundary. Other typical probes are Si with a Pt/Ir coating [55] or with a chromium buffer layer followed by gold [56]. For measurements on molecular wires by CP-AFM, Ishida et al. reported apparently negative differential resistance values at a higher bias which they attributed to the surface roughness, and a strong influence of the tip-molecule contact on the carrier transport through the system [57].

Concluding, Table 1 gives a brief overview of the similarities and differences of the AFM modes mentioned in this section.

## 3. Biopolymers and Hydrogels

The aforementioned methods can be used on a broad variety of biopolymers and hydrogels diversely applied due to their biodegradable and biocompatible properties in agriculture, pharmaceutical, biomedical, food, and cosmetics due to their biodegradable and biocompatible properties. The biopolymers widely applied in these biotechnological applications such as chitosans, celluloses, carrageenans, alginate, polyesters, enzymes and DNA are broadly classified into polysaccharides, polynucleotides and polypeptides [58]. For the purpose of this review, some of the most interesting biopolymers in the form of films as well as hydrogels are presented here in brief.

### 3.1. Carrageenan

**Figure 8:**
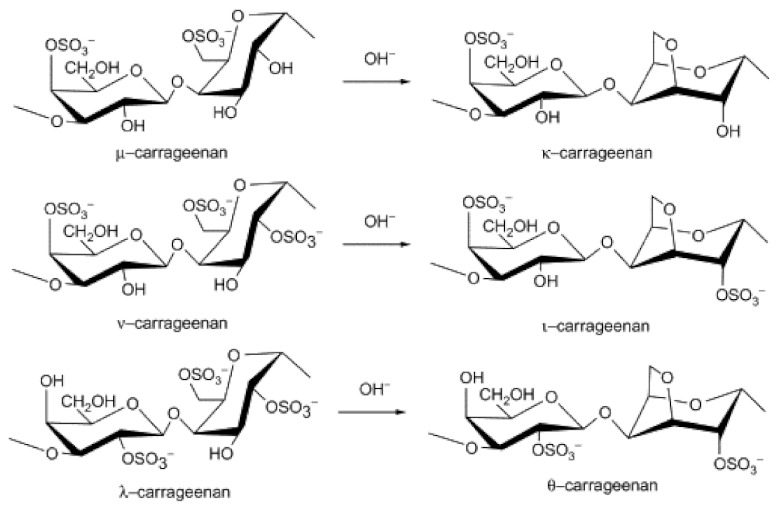
Structures of carrageenans. Reprinted from [61]. Copyright (2014), with permission from Elsevier.

Carrageenans are sulfated galactans extracted from red seaweeds and are classified as κ-, ι-, and λ-carrageenans. They are linear chains of alternating 3-O-substituted β-d-galactopyranosyl units and 4-O-substituted α-d-galactopyranosyl units. κ- and ι-type carrageenans differentiate from other types by an internal 3,6 ether bond. Sugar units have one or two sulfate groups esterified to a hydroxyl group at the carbon atoms C2 or C6 in all types of carrageenans [59]. The carrageenan content of commercial seaweeds is normally 30–60% of dry weight and is located in the cell wall and the intercellular matrix of the plant tissue. Carrageenan is sourced mainly from *Kappaphycus alvarezii* and *Eucheuma denticulatum* [60]. The structure of different carrageenans is depicted in Figure 8 [61].

Carrageenans have a wide range of applications such as being an emulsifier, stabilizer, or thickeners for food applications, in shampoos and cosmetics, cell immobilization, lubricants, etc. The applications depend upon the macromolecular structure of the hybrid polysaccharides and their concentration. Kappa carrageenan forms strong, rigid gels in the presence of potassium ions [62]. Iota carrageenan forms soft gels in the presence of calcium ions while Lambda carrageenan does not gel. Single and double stranded structures are seen for κ-Carrageenan at low concentrations. Fibrous network-like structures by a side-by-side association are seen in κ-, κ/β-, and κ/ι-carrageenans at high concentrations, end-to-end association is seen in κ/ι-carrageenan. Open networks with coarser fibers are formed by κ/β-carrageenan while the κ/ι-carrageenan structure forms a more flexible network. X-carrageenan presents with honeycombed structures due to end-to-end and side-by-side association types, while λ-carrageenan forms honeycombed structures only at high concentrations [63]. Atomic force microscopy can provide insight into these topographical parameters of the carrageenan’s macromolecular structure which can be further investigated for its applications.

### 3.2. Chitosan

Chitosans are linear polymers produced by N-deacytelation of chitin and are made up of 2 sub-units, D-glucosamine lined to N-acetyl-D-glucosamine by 1,4 glycosidic bonds [64]. Their sources in nature vary from crustaceans like lobsters, crabs, etc., to insects like spiders, cockroaches, beetles and many microorganisms such as fungi and algae [65,66]. Chitosan has a large set of applications, in agriculture, food, cosmetic, textile, and biomedical industries [67,68]. Chitosan being mucoadhesive, biodegradable, biocompatible and hydrophilic has been recently gaining attention for drug releasing properties in medicinal and nanomedical applications [69]. This drug release is dependent on the average molecular weight of chitosan and increasing its porosity [69]. The effect of various factors on protein release from alginate–chitosan coacervate microcapsules include the alginate, chitosan and calcium chloride concentration, loading rate, chitosan molecular mass and pH of the gelation medium as reported by Vandemberg et al. [70]. AFM is becoming increasing established as a tool for characterizing the drug delivery vehicles and target action of nanoparticles. AFM has been used for confirming the shape of differently shaped chitosan nanoparticles, size, surface roughness and liposomal drug deliveries [71]. Additionally, chitosan belongs to the materials which are often used in electrospinning, in this way forming nanofibers similar to those depicted in Figure 4 [72,73,74].

### 3.3. Alginate

Alginic acids are natural polysaccharides extracted from seaweeds. They consist of different ratios of beta-D-mannuronic acid and alpha-L-guluronic acids, linked through 1-4-glycosidic bonds [75]. Gelation is induced by the addition of divalent cations, most commonly calcium ions. The cations form ionic bonds with the sequences of the polymer that are high in guluronic acids resulting in “egg-box” shaped structures [76]. Consequently, the strength of the hydrogel alginate depends on the concentration of guluronic acids, of the polymer itself and of the divalent ions as well as on the choice of divalent ions [76,77,78]. Alginate hydrogels are reported to be hydrophilic, biocompatible, biodegradable, non-toxic and easy to gel. For this reason, they are employed for a variety of entrapment purposes including agricultural applications [79,80,81], wastewater treatments [82,83], food additives [84], heterogeneous catalysis [85,86,87,88], and biomedicine [84,89].

With atomic force microscope, the roughness [90,91,92,93,94], size and shape [95,96,97,98], structure [99], membrane adhesion (fouling) [91,100,101], surface electrical properties [91] and elasticity modulus [102] of alginate hydrogels are studied.

### 3.4. Silica Hydrogels

Silica hydrogels are prepared by sol-gel synthesis from various precursors. Due to their inorganic structure, they display a higher resistance against biodegradability compared to biopolymer hydrogels. The biocompatibility of the hydrogels depends on the chosen precursor and the employed preparation method. In addition to conventional aqueous and alkoxide precursors like sodium silicate, tetraethyl orthosilicate and tetramethyl orthosilicate, functionalized precursors are employed in order to modify the hydrogels for the application. Silica hydrogels are applied for the entrapment of enzymes and cells for heterogeneous catalysis [103,104,105,106], biomedicine [107,108], environmental remediation [108], biosensors [109,110] and biofuel cells [111]. For more detailed reviews see [110,112].

With atomic force microscopy, the adhesion forces between silica surfaces and other hydrogels or cells [113,114,115,116] and the friction force [116], the mechanic and visco-elastic properties [117,118], the surface roughness [119], the morphology and size of nanoparticles [120,121,122,123,124,125] and the uptake of nanoparticles by cells [121] were studied.

## 4. Applications

AFM measurements on biopolymers and hydrogels can be straightforward in the case of rigid polymers with even surfaces but can also cause challenges for sample preparation. The next section thus introduces a few typical sample preparation techniques, before presenting examples of AFM measurements on different biopolymers as films and hydrogels.

### 4.1. Sample Preparation

As mentioned before, AFM investigations on extremely soft samples are usually performed in the tapping or non-contact mode to minimize sample damage. In the case of lubricating greases, Roman et al. underline the importance of producing an even surface, in this case by heating the sample to allow the surface to flatten and measuring after the sample has cooled down to room temperature [126].

A typical method to receive a flat surface of a biopolymer is aerosol spraying of an aqueous solution onto a freshly cleaved mica substrate and letting it dry by air. McIntire and Brant described this method for the AFM measurements of different biopolymers and supramolecular assemblies and showed linear and cyclic triple helices of the polysaccharide scleroglucan, the contour length and chain thickness in xanthan, the formation of stiff fibrillar gellan aggregates, a single-stranded state of κ-carrageenan, etc. [127]. In a previous paper, the group mentions that the forces between the cantilever tip and the biopolymer are in the same order as those between the polymer and the mica substrate, making further fixation of the biopolymer unnecessary [128].

A potential problem of this sample preparation method, however, is mentioned by Baalousha and Lead who report about possible formation or artifacts, such as aggregation and salt crystallization if the sample is not sufficiently washed after drying [129]. Klinov et al. also mention a washing step after dropping the DNA solution on mica, followed by drying the sample with compressed argon [130].

Flamia et al. reported different behavior of an elastin-like biopolymer depending on the deposition either from methanolic or aqueous suspensions, with this polypeptide evolving from layers to ribbons to beaded filaments in methanol, while it self-assembled in fibrillar networks or in amyloid-like patterns in water [131].

Similarly, Morris et al. showed in the case of xanthan that depending on the preparation, a solution or a microgel may be produced, resulting in the possible misinterpretations of AFM images [10].

A study by Balnois and Wilkinson compared drop deposition with ultracentrifugation and deposition by adsorption form a liquid suspension in the case of the polysaccharide schizophyllan on mica [132]. They found that aggregates of different dimensions were formed, with ultracentrifugation giving the largest aggregates and adsorption resulting in only a few aggregates.

Finally, another approach should be mentioned, reported by Matsko and Mueller who embedded biological material in resin as a preparation for AFM images [133]. They used high-pressure frozen and in acetone freeze-substituted nematodes which were warmed up again before embedding them in resin mixtures. Infiltration was performed in different steps, starting with 33% resin in acetone, 66% resin in acetone, and finally 100% resin in a desiccator to work under dry conditions, before the samples were polymerized at 60 °C. These samples could then be cut intro ultrathin sections using a microtome. They found a dependence of the surface topography on the epoxy hardness and showed that the ethanol treatment of the block face after microtome slicing made small details visible.

### 4.2. Carrageenan

As described in Section 3.1, different carrageenans exist with different macroscopic structures. λ-carrageenan has a linear structure which was investigated by Diener et al. in the presence of chloroquine, an ionic drug normally used against malaria [134]. With topographical image analysis by the AFM, they found that the single polysaccharide chains increased in height upon the addition of chloroquine, which was attributed to the formation of a secondary structure and subsequent higher hierarchical aggregates. These new forms disappeared again when inorganic cations such as Na^+^ replaced the chloroquine.

McIntire and Brant showed the single-stranded state of κ-carrageenan in the form of well-separated polymer chains with heights of less than 1 nm [127].

For the polyelectrolyte complex of carrageenan and chitosan, AFM measurements revealed that the chitosan was positioned on the carrageenan fiber surface [135]. In mixtures dominated with carrageenan, the chitosan was incorporated in the carrageenan network instead.

**Figure 9:**
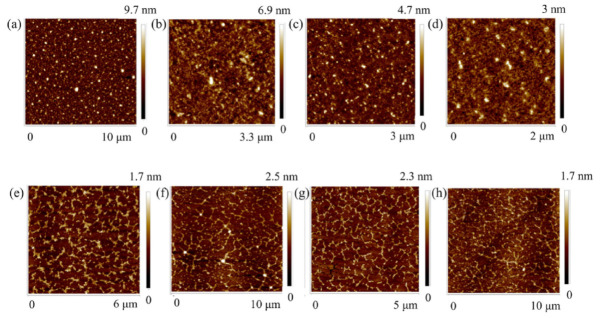
AFM images of polysaccharide extracts from *Euchema*, mostly containing ι-carrageenan. Extraction conditions/temperatures were: (**a**) 0.02 mg/mL 60 °C extract; (**b**) 0.02 mg/mL 70 °C extract; (**c**) 0.02 mg/mL 80 °C extract; (**d**) 0.02 mg/mL 90 °C extract; (**e**) 0.002 mg/mL 60 °C extract; (**f**) 0.002 mg/mL 70 °C; (**g**) 0.002 mg/mL 80 °C extract; and (**h**) 0.002 mg/mL 90 °C. Reprinted from [136]. Copyright (2020), with permission from Elsevier.

Yang and Yang investigated the temperature dependence of the extraction of polysaccharides from *Euchema* for temperatures between 60 °C and 90 °C [136]. They found a lower molecular weight and reduced layer height for higher extraction temperatures which was advantageous in terms of the gelling potential, while lower temperatures resulted in more diverse polysaccharides. The main component was ι-carrageenan. Figure 9 shows the AFM topography images of the different extracts, diluted with deionized water in different concentrations. Larger heights were attributed to side-by-side association of the polysaccharides.

A mixture of κ- and λ-carrageenan with a high molecular weight was used by Webber et al. who prepared carrageenan/chitosan multilayers in which they partly embedded nisin Z [137]. They applied the peak force tapping mode in a quartz liquid cell with 0.1 M KCl at ambient temperature to measure topography and the sample thickness on a silicon waiver by scratching the sample with a stainless-steel needle. They found comparable heights for the samples with and without nisin Z and the highest roughness for the sample without nisin Z. Similar κ-carrageenan/chitosan multilayer films were produced by Zhuikova et al. who saw a change in the root mean square roughness with the number of layers [138]. For a carrageenan/guar gum blend, Swain and Bal used AFM to measure the surface roughness depending on in-vitro degradation studies [139]. Roy et al. added carbon dots to carrageenan/gelatin films for food packaging and found a reduced surface roughness for most combinations with carbon dots as compared to the pure carrageenan/gelatin films [140]. Muthulakshmi et al. used AFM topography images to show the improved corrosion resistance of a medical-grade stainless steel in mild acidic conditions due to a carrageenan/gelatin coating [141]. Similarly, Souza et al. investigated the topography of a κ-carrageenan multilayer coating with quercetin-loaded lecithin/chitosan nanoparticles and found a higher roughness for the quercetin nanoparticle layers than for the κ-carrageenan layers [142].

Only a few papers report about phase imaging used on carrageenan blend films to detect the material composition on the surface [143]. Nanoindentation experiments have been scarcely used to investigate the mechanical properties of ι- and λ-carrageenan films [144] or on nanotubes from bovine serum albumin with κ-carrageenan [145].

PeakForce QNM measurements on carrageenan can be found in some papers. Schefer et al. report on measurements evaluated according to the Derjaguin–Muller–Toporov (DMT) model on κ-carrageenan to measure the elastic modulus for a thick film in the presence of 100 mM KCl as well as on a network of superstructures, the latter showing a bimodal modulus distribution [146]. Simkovic et al. showed peak force modulus, stiffness and adhesion maps for ι- and κ-carrageenan and many other biopolymers [147].

No investigations of systems containing carrageenan using KPFM or CP-AFM were found in the literature.

### 4.3. Chitosan

Chitosan samples are also most often investigated by AFM to measure their topography or the surface roughness [148,149,150,151,152]. Matienzo and Winnacker investigated the influence of oxygen plasma and UV/ozone irradiation of chitosan films and found a much smoother surface for the latter, as compared to the harsher conditions during oxygen plasma treatment [153]. Sionkowska et al. measured the surface roughness of different chitosan blends by AFM [154].

Laser-induced periodic surface structures in chitosan, starch and chitosan/PVP blends were investigated by AFM [155]. Pérez et al. showed that the formation of such periodic surface structures was possible in the amorphous chitosan and chitosan/PVP films, while the crystalline starch film could not be modified in this way.

**Figure 10:**
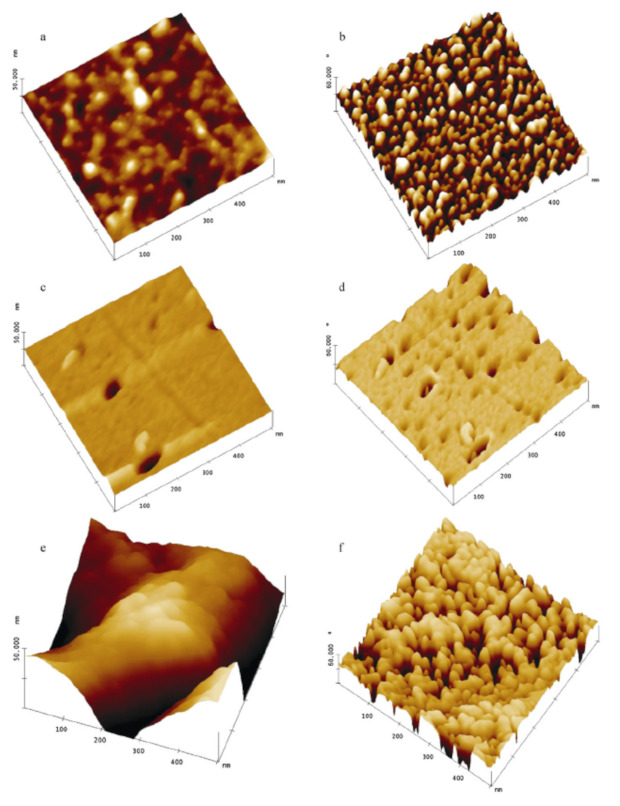
AFM images (500 nm × 500 nm) of the reference sample (**a**) morphology and (**b**) phase image; the same sample containing Zn^2+^ ions, after adsorption from the Zn^2+^ ion solution (initial concentration 80 mg/L) (**c**) morphology and (**d**) phase image; (**e**) the same sample containing Zn^2+^ ions adsorbed (initial concentration 320 mg/L) and (**f**) the phase image. For surface morphology images z-range is 10 nm, while for phase images z-range it is 60°. Reprinted from [156]. Copyright (2011), with permission from Elsevier.

Besides these topography-related measurements, phase imaging is reported more often on chitosan than on carrageenan. Milosavljevic et al. used phase imaging to show chitosan with and without Zn^2+^ ions, using this method to enhance the edges and thus to highlight particular grains, as visible in Figure 10 [156]. Similar effects were found for Cu ions loaded on chitosan/itaconic acid hydrogels [157].

For chitosan, similar to carrageenan, some PeakForce QNM measurements can be found in the literature. Gaihre and Jayasuriya report on different chitosan composite scaffolds on which PeakForce QNM were performed to investigate their nanomechanical properties by DMT calculation [158]. They used an antimony-doped Si cantilever which was suitable for typical chitosan Young’s moduli and found an increased modulus for all blends in comparison with pure chitosan. Similarly, Luna et al. investigated the nanomechanical properties of chitosan films as a function of temperature and pH by peak-force AFM [159], and Mendes et al. mapped the stiffness of electrospun chitosan/phospholipid hybrid nanofibers by this method [160].

Contrary to carrageenan, chitosan investigations by KPFM are reported a few times in the literature. Rocha Neto et al. used this method to measure the surface potential of multilayered chitosan/hyaluronan coatings [161]. KPFM images of chitosan after the application of positive and negative DC pulsed fields showed larger areas with trapped charges for negative than for positive pulsed fields [162]. Hernandez-Montelongo et al. measured the surface potential of the step from a nanofilm to the Si substrate for 3 and 9 hyaluronan/chitosan bilayers and found the surface potential step equivalent to the height step [163].

### 4.4. Alginate

AFM on alginate is again mostly related to morphological investigations. Singh et al. used AFM to measure the topography of an alginate/sterculia gum based hydrogel which they suggested as a possible material for brain drug delivery [164], while Aburabie et al. found that the roughness of the substrate transferred onto the coated alginate membranes which they proposed for organic solvent nanofiltration [165]. Badita et al. reported a significant increase in surface roughness upon crosslinking sodium alginate with Ca^2+^, as shown by AFM topography maps [166]. Zhang et al. investigated in detail the grooves on alginate microfibers prepared by microfluidic spinning in different concentrations of alginate in deionized water [167]. Alginate/chitosan/alginate-modified silica nanocapsules and chitosan multilayer films were investigated during pH-triggered swelling and deswelling, and topography measurements by AFM were used to verify that their structural integrity in PBS was maintained [168].

**Figure 11:**
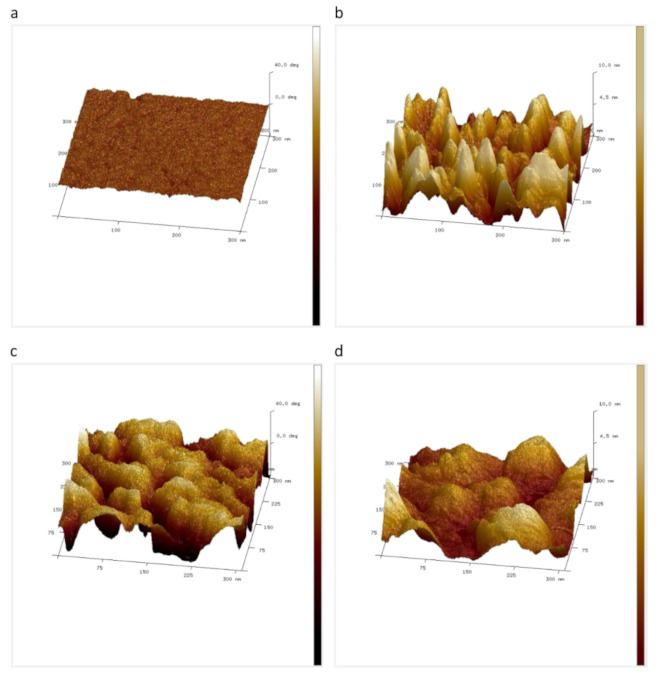
AFM images of alginate films. (**a**) phase image and (**b**) topography image of pure alginate film; (**c**) phase image and (**d**) topography image of alginate films with chlorhexidine hexametaphosphate particles. Lateral image dimensions are 300 nm; the *z*-axis represents 40° (phase images) and 10 nm (topography), respectively. Reprinted from [169], originally published under a CC-BY license.

Moreover, some papers report about phase imaging to investigate alginate samples. Duckworth et al. compared topography and phase images of alginate films with chlorhexidine hexametaphosphate particles as well as pure alginate films, as depicted in Figure 11 [169]. Opposite to Figure 10, the authors here found a high roughness in both cases (Figure 11b,d), but only for the alginate film with chlorhexidine hexametaphosphate particles did they also find variations in stiffness, leading to variations in the phase image, while the phase image of the pure alginate film was quite even. Axpe et al., on the other hand, used phase imaging to increase the edge contrasts of alginate nanocomposites [170].

Nanoindentation was used by several groups to investigate alginate samples’ elastic modulus [170,171,172], while others used PeakForce QNM mapping on alginate capsules for this purpose [173]. A few groups report about the KPFM measurements, e.g., for single alginate molecules adsorbed on hematite and other iron oxides [174] or of silica sensors coated with alginate [175].

### 4.5. Silica Hydrogels

Similar to the aforementioned biopolymers, AFM investigations on silica hydrogels mostly aimed at surface investigations. Betiha et al. produced ionic liquid-cellulose-silica hydrogels and showed the surfaces of pure methylcellulose and the different nanocomposites [176]. Carvalho dos Santos et al. prepared silica hydrogels with poly(melamine-formaldehyde) (PMF) and used AFM images for surface investigations [177]. Silica hydrogels and aerogels were examined by AFM topography measurements as well as by PeakForce QNM [178]. Jiang et al. showed AFM images of silica hydrogels with carbon nanofibers to monitor the aging of the samples [179].

Becerra et al. prepared fibrin hydrogels with silica or chitosan/silica and used AFM in the force spectroscopy mode to measure Young’s modulus, showing the increase in stiffness due to the addition of silica [180]. Silica aerogel was prepared from silica hydrogel, and the surface topography was investigated by AFM [181]. Silica hydrogels were investigated by different mechanical experiments based on an interaction with a cantilever with a silica colloid attached [182].

Besides these topography and mechanical measurements, no KPFM or CP-AFM measurements on silica hydrogels were found in the literature.

### 4.6. Biopolymer Hydrogels

Besides silica hydrogels, there are also biopolymer hydrogels which were investigated by AFM. The morphological structure of poly(vinyl alcohol) (PVA) films with different amounts of κ-carrageenan hydrogel films, e.g., were investigated by AFM, showing a reduced roughness with increasing amount of κ-carrageenan [183]. For κ-carrageenan/gelatin blend hydrogels prepared for tissue engineering, AFM was also used to investigate the surface morphology [184]. Similarly, the texture of κ-carrageenan hydrogels for vegan gummy candies was examined by AFM [185].

Reports about the AFM measurements on chitosan hydrogels can be found much more often in the literature. Besides the morphological investigations [186,187,188], there are also investigations of the mechanical properties of chitosan hydrogels using force-distance curves to evaluate the elastic modulus [189] as well as PeakForce QNM measurements [190]. Phase images were used to investigate the co-existence of two phases in chitosan/carboxymethylcellulose hydrogels [191]. KPFM and CP-AFM measurements, however, are scarcely reported in the literature.

For alginate hydrogels, again surface images and roughness were investigated by AFM [192] as well as the elastic modulus, e.g., of soft calcium-alginate hydrogels with an elastic modulus in the range of 100–4500 Pa [193]. KPFM was measured on alginate hydrogels used for drug delivery, showing that the drug layer was nearly neutral with a surface potential of 7 mV [194].

As these limited examples show, AFM on these and diverse other biopolymer hydrogels is also quite common in the literature; however, typically simple surface topographies are investigated. Comparatively, other modes may offer further relevant information, often without too much additional effort.

### 4.7. Other Biopolymers and Hydrogels

At the end of this section, Table 2 gives a brief overview of additional biopolymers and hydrogels about which interesting AFM measurements are reported in the literature, to give an idea of the universal applicability of this technique for biopolymers and hydrogels.

It should be mentioned that while here many possible measurements are reported, there are also limitations of AFM measurements on biopolymers and hydrogels. These are mostly related to the rough surface which can occur for many sample preparation methods, as well as to the extremely soft materials which are often involved. While rough surfaces limit the possible resolution or even impede measurements by breaking the tip at abrupt height increases, soft materials can themselves be erroneously modified or destroyed by sharp tips, especially in the contact mode. Biopolymers like alginate and carrageenan shrink in contact with the atmosphere due to dehydration. This is particularly an issue for dry measurements of the biopolymers and additional time consuming sample preparation is required to analyze these biopolymers in wet mode. These potential problems have to be taken into account and necessitate a preliminary understanding of the sample’s topography and its mechanical properties to define the suitable measurement parameters and choose the optimal tip material and dimensions.

## 5. Conclusions

AFM has become a frequently used tool for the investigation of biopolymers and hydrogels. While mostly the surface topography and the roughness are examined, especially in the phase imaging mode—which is integrated in modern AFMs if the tapping mode is used—was unexpectedly found only scarcely, although it offers the possibility to sharpen edges and to receive qualitative information about the material composition in the sample.

Besides the topography, some attempts can be found in the literature to measure the mechanical properties, either by PeakForce QNM or by nanoindentation. All electrical modes are used scarcely on biopolymers and hydrogels.

Our study shows that not only the more complicated modes which necessitate special cantilevers and sometimes sophisticated sample preparation, but especially the simple phase imaging mode offers in many cases significantly more information than the pure topography image and should, thus, be used more often by researchers working on biopolymers, hydrogels and other biological samples. This review aims at contributing to broadening the range of AFM modes used on such samples in the future.

## Figures and Tables

**Table 1:** Characteristics of different AFM modes.

AFM Mode	Information Given	Static/Dynamic Mode	Special Requirements
Contact mode	Topography, roughness	Static mode	Cantilever for static mode
Tapping mode	Topography, roughness	Dynamic mode	Cantilever for dynamic mode
Phase imaging	Qualitative differentiation between materials + clearer edges within single material	Dynamic mode	Cantilever for dynamic mode
Nanoindentation	Elastic modulus, hardness	Dynamic/static/quasistatic	Tip harder than sample
PeakForce QNM	Elastic modulus, adhesion	Dynamic mode	Often very broad tips are supportive, functionalization of the cantilever broadens the measurement spectrum
KPFM	Contact potential (work function) of a surface	Dynamic mode	Conductive tip, single or dual pass setup
Conductive AFM	Conductivity; local current-voltage curves	Static mode	Conductive tip

**Table 2:** Examples of other biopolymers and hydrogels, investigated by AFM.

Material	Properties Measured by AFM	Reference
Agarose-polyethylene glycol-polycaprolactone gel	Topography and nano-phase separation	[195]
Polysaccharide-polycaprolactone gel		[196]
Agarose/polycaprolactone		[197]
Poly(ethylene glycol)/poly(caprolactone)		[198]
Bacterial biopolymers	Adhesion between biopolymer and SiN tip	[199]
Starch	Mechanical properties by PeakForce AFM	[200]
Starch and gluten	Nano-mechanical properties	[201]
Biopolymer-doped polypyrrole	Adhesion, E-modulus, electrostatic force	[202]
Polysaccharides on bacterial cells	Stretching biopolymer molecules for force-extension tests	[203]
Chitosan hydrogels	Topography	[204]
Caffeine in polymer matrix	Topography and phase imaging	[205]
Pectin-rich biopolymer from citrus waste	Topography	[206]
Hexaglycylamide		[207]
Hydrogels and cells	Indentation with different tip shapes	[208]
Soft hydrogels	Test of microindentation parameters	[209]
Gelatin	Nanoindentation	[210]
Gelatin nanofibers	Morphology	[211]
Chitosan/hydroxyapatite/nanoZrO_2_	Roughness of laser-structured surfaces	[212]
Fibrin hydrogel with gold nanowires	Adhesion force, stiffness, elasticity	[213]

## Data Availability

Not applicable.
